# Historical Facts of Acupuncture and Traditional Chinese Veterinary Medicine—A Letter to the Editor Re: Magalhães-Sant’Ana, M. *Animals* 2019, *9*, 168

**DOI:** 10.3390/ani10071196

**Published:** 2020-07-15

**Authors:** Yusheng Hu, Zhongjie Liu

**Affiliations:** College of Veterinary Medicine, China Agricultural University, Beijing 100193, China; hys_tcvm@cau.edu.cn

**Keywords:** traditional Chinese veterinary medicine, acupuncture, bloodletting, history

## Abstract

A recent viewpoint paper by Manuel Magalhães-Sant’Ana (2019) discussed the evidence regarding history, conceptions and modern research related to Traditional Chinese Veterinary Acupuncture (TCVA). Based on the observation of an illustration of nine needles, the author suggested that the needles used in acupuncture are more like lancets than needles in ancient times; to support the view that acupuncture is analogous to bloodletting. In addition; the author does not believe that TCVA has not been practiced for thousands of years. This letter documents that the prototype of the modern filiform acupuncture needle has appeared as early as the Han Dynasty and that modern needles did not evolve from lancets. In addition, there is proof based on existing ancient books that TCVA has a history of thousands of years.

Dear Editor,

Manuel Magalhães-Sant’Ana in his critique of Traditional Chinese Veterinary Acupuncture (TCVA), discussed the evidence regarding history, conceptions and modern research related to TCVA. With the so-called “epistemological approach”, he asserted that the TCVA is analogous to bloodletting [1]. Based on our basic knowledge and acupuncture clinical experience, we believe that the author’s interpretation of the TCVA historical evidences is incorrect and does not support his claims. 

Based on the observation of only one nine-needle illustration, the author states that the needles should be described as lancets rather than needles, and, on this basis, the author concludes that acupuncture is similar to bloodletting. The concept of nine-needle first appeared in *Huang Di Nei Jing* [黄帝内经, The Yellow Emperor’s Inner Classic of Medicine] (HDNJ), where the name, form and main treatment of nine-needles were discussed in detail [2]; however, there was no illustration of the nine needles. Since the Yuan Dynasty, there have been scholars in all dynasties who have shown illustrations of the nine needles based on the records from HDNJ. Until today, there are basically two painting styles regarding the shape of the needles. The illustration cited by the author (Figure 1A), was drawn by Yang Jizhou [杨继洲]. Yang’s work was based on a similar illustration (Figure 1B), which was drawn by Xu Chunfu [徐春甫] in the Ming Dynasty, and was slightly modified. Another style is represented in Figure 1C, which is the earliest existing nine-needle illustration [2]. Any reader who only looks at Figure 1A will find that four needles (1, 5, 6, and 9) look like lancets, but this impression is largely due to the influence of the painting style. If Magalhaes-Sant’Ana would have seen the nine-needle illustration from *Zhen Jiu Zhai Ying Ji* [针灸摘英集, Collection of Acupuncture and Moxibustion Digests] and the modern nine needle model (Figure 1C,D), in which only two out of nine (#5 and #9) look like lancets, he might agree that most of them could not be described as lancets.

It is obviously not correct to only judge by the illustration alone because of the influence of the painting style. Therefore, it is more appropriate to infer the shape of the nine needles according to the descriptions of their shape and details of their use. All illustrations were drawn according to the description from the HDNJ, so the captions in different illustrations were relatively consistent. Through these captions, we can divide these nine needles into three categories: in the first one, the needles are slender, thin and long, similar to today’s acupuncture needles, including Huozhen [火针] (#1 in Figure 1), Changzhen [长针] (#2, Figure 1), Haozhen [毫针] (#3, Figure 1), and Yuanlizhen [员利针] (#4, Figure 1). Their main function is to puncture the body. The second category is massage equipment, including Dizhen [鍉针] (#7, Figure 1) and Yuanzhen [员针] (#8, Figure 1), which has no sharp point, and is mainly used for pressing and rubbing the body surface. The third type is similar to the lancet, including Pizhen [铍针] (#5, Figure 1), Fengzhen [锋针] (#6, Figure 1) and Chanzhen [镵针] (#9, Figure 1). Among these three needle types, Fengzhen and Chanzhen were mainly used for bloodletting. Among the nine kinds of needles, only two are mainly used for bloodletting, and more than half (6/9) of them are different in appearance from the lancet. The author’s conclusion “most of them would be better described as lancets, similar to those used by European barber surgeons, rather than needles” therefore appears incorrect. In addition, from the HDNJ [4], we know that modern acupuncture technology and the needles had their origin in the Han Dynasty. Therefore, there is no support for the author’s statement that the modern needle came from the improvement of the lancet. The author also quoted the description of bloodletting therapy from HDNJ and ancient Korean veterinary books [5,6] in the attempt to prove that needling was used for bloodletting. Indeed, there is a therapy associated with bleeding, which is called Xuezhen [血针, Hemoacupuncture] [7]. However, as one of many acupuncture treatments, Xuezhen is far less widely used than Baizhen [白针, filiform needle acupuncture] and Dianzhen [电针, electro acupuncture]. 

The author stated that the TCVA does not have a thousand-year history. Because we are unable to access the full text of the literature cited by the author, we are not sure what the basis of the author’s argument might be. However, there is written evidence that the TCVA has been used for thousands of years. *Si Mu An Ji Ji* [司牧安骥集, Collection on the Treatment of Equine diseases] (SMAJJ) is the earliest and relatively complete veterinary work in China [8]. Its content mainly describes the treatment of equine diseases, with many references concerning acupuncture (PDF1 in Supplementary Materials) [9]. The date of completion of SMAJJ is about 838 A.D., 1183 years ago [10]. In addition, acupuncture was also mentioned in the bamboo clip book of *Yi Ma Shu* [医马书, Equine Veterinary Manual] from Lao Guan Shan Tomb of Han Dynasty (202 B.C.-8 A.D.) in Chengdu in 2012 [11]. In his critique, the author mentioned that there was no reference to acupuncture in Yuan Heng Liao Ma Ji [元亨疗马集, Yuan and Heng’s Collection on the Treatment of Equine diseases] (YHLMJ). However, the chapters *Bo Le Ming Tang Lun* [伯乐明堂论], *Liu Mai Ming Tang Ge* [六脉明堂歌] and *Lun Ma Ming Tang Zhen Xue Zhe He Ye* [论马明堂针穴者何也] in YHLMJ are devoted to the application of acupuncture in the treatment of equine diseases (PDF2, PDF3, PDF4 in Supplementary Materials) [12]. Similarly, the author’s statement that the charts in YHLMJ are mainly used to indicate the anatomic location of pathologic conditions or points for cauterization and bleeding appears incorrect [13,14]. Figure 2 is an illustration from chapter Bo Le Ming Tang Lun, which shows that the acupoints were used in the treatment with white needles and fire needles (a method of TCVA) [15].

The interpretation of the hair whorls illustration (Figure 3A) is also incorrect. This picture was used to show the good and ill luck represented by different hair whorls in the ancient concept. The captions in this picture were not to describe the horse’s temperament as stated by the author, but the name of each hair whorl. In YHLMJ printed in 1990 [16], we can find the same pictures with clearer captions (Figure 3B). In each caption, the first two characters indicated the location of the hair whorl, and the second two characters represented the judgment of good or ill luck. 

In conclusion, extensive knowledge of the literature and careful interpretation of its findings leads us to believe that TCVA and Galen’s bloodletting therapy are very different from theory to practical application [17,18,19]. 

The time frame of TCVA has been applied in the treatment of small animal diseases is much shorter than the thousand-year history of that of equine diseases [20]. Although the theories and methods of acupuncture from human and equine medicine can be used for reference, we agree that more clinical studies and high-quality research are still needed to promote the application and development of TCVA in small animal medicine. Advanced research of the effect of TCVA in canine and feline diseases should be fully encouraged. Furthermore, we must realize the fact that, due to the limitation of language, most of the literature research on TCVA has not involved the Chinese database [21,22]. However, the number of studies and clinical reports on TCVA in the Chinese database is so large that neglecting them will affect the accuracy of the conclusion.

## Figures and Tables

**Figure 1:**
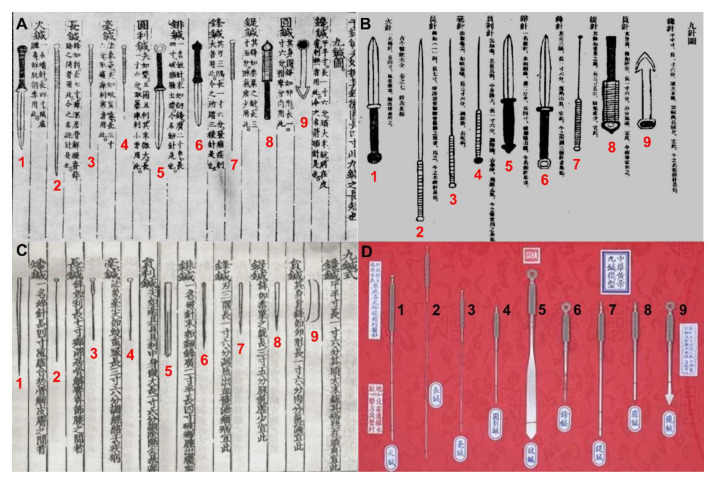
Nine-needle illustrations and model. (**A**) Illustration of nine needles from *Zhen Jiu Da Cheng* [针灸大成, Great Compendium of Acupuncture and Moxibustion] [1]. (**B**) Illustration of nine needles in *Gu Jin Yi Tong Da Quan* [古今医统大全, Great Collection of Ancient and Modern Medicine] [2]. (**C**) Illustration of nine needles from *Zhen Jiu Zhai Ying Ji* [针灸摘英集, Collection of Acupuncture and Moxibustion Digests] [2]. (**D**) Modern nine-needle model made according to the description of nine- needles from *Huang Di Nei Jing* [黄帝内经, The Yellow Emperor’s Inner Classic of Medicine] [3]. The same number in each figure represents the same kind of needle.

**Figure 2:**
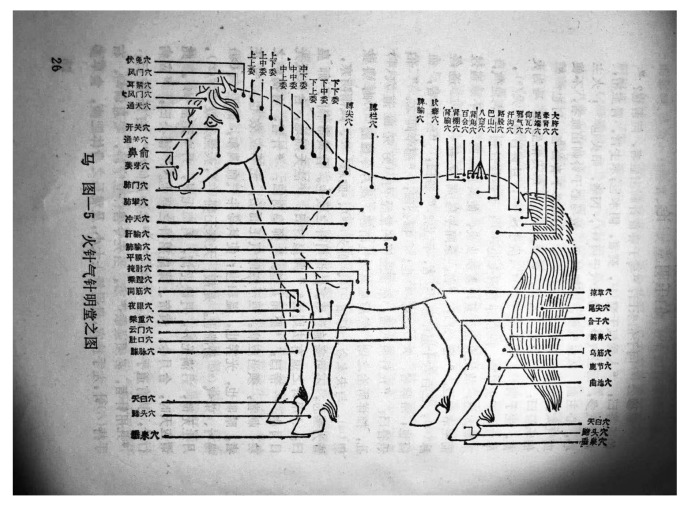
Illustration of the acupoints in *Bo Le Ming Tang Lun* [15]. One of the chapters in *Yuan Heng Liao Ma Ji*, *Bo Le Ming Tang Lun* was devoted to the application of acupuncture in the treatment of equine diseases.

**Figure 3:**
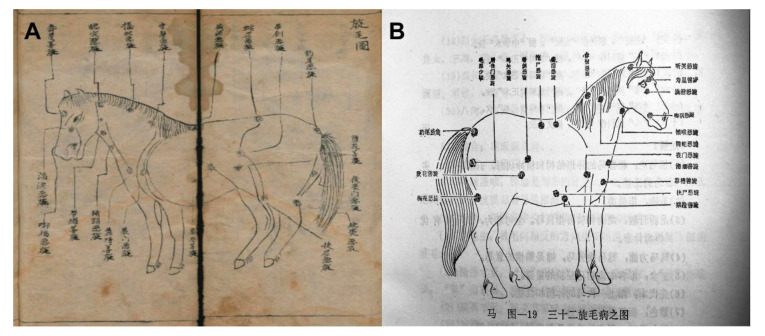
Different versions of the hair whorls illustration. (**A**) The illustration of hair whorls quoted by the author. (**B**) The illustration of hair whorls in *Yuan Heng Liao Ma Ji* printed in 1990 [16].

## Supplementary Materials

The following are available online at https://www.mdpi.com/2076-2615/10/7/1196/s1, PDF 1, PDF 2, PDF 3 and PDF 4.
