# Supplementary material for: Historical Facts of Acupuncture and Traditional Chinese Veterinary Medicine—A Letter to the Editor Re: Magalhães-Sant’Ana, M. Animals 2019, 9, 168
Source: Animals (Basel). 2020 Jul 15;10(7):1196. doi: 10.3390/ani10071196 (PMC7401525; doi:10.3390/ani10071196)
Supplement: Supplementary file 1 [file animals-10-01196-s001.zip › Supplementary materials/PDF1 Bo Le Zhen Jing.pdf]

司牧安骥集校注

唐·李　石等　编著
邹介正　和文龙　校注
李　群　陈少华　王铭农　参校

中国农业出版社

图书在版编目（CIP）数据

司牧安骥集校注／（唐）李石等编著；邹介正，和文龙校注．—北京：中国农业出版社，2001.11
ISBN 7-109-07071-9

Ⅰ.司...　Ⅱ.①李...②邹...③和...　Ⅲ.①马—饲养管理②司牧安骥集—注释　Ⅳ.S821

中国版本图书馆 CIP 数据核字（2001）第 053834 号

中国农业出版社出版
（北京市朝阳区农展馆北路 2 号）
（邮政编码 100026）
出版人：沈镇昭
责任编辑　白洪信

中国农业出版社印刷厂印刷　新华书店北京发行所发行
2001 年 12 月第 1 版　2001 年 12 月北京第 1 次印刷

开本：850mm×1168mm 1/32　印张：13.75
字数：350 千字　印数：1～1 000 册
定价：48.50 元
（凡本版图书出现印刷、装订错误，请向出版社发行部调换）

伯乐针经[1]

大凡行针，先知穴道去处，次辨浅深补泻之法，免其失误。右手持针，左手按穴。行针切忌大雨大风；缘风是祸害，雨是绝命，阴阳纷争，不可行针。用针须依穴道，看病浅深，补泻相应；出气为虚，入气为实；左转针为补。右转针为泻；后按针为中补，次后针为中泻。若偏较一丝，不如不针，隔一毫如隔泰山，仔细审详，对病行针，何愁不痊，具穴道如后：

眼脉穴：在眼后四指[2]。是穴入针二分，出血。疗肝脏热、眼肿、泪下病。

鹘脉穴：在颊骨下四指[3]。是穴入针三分，出血。疗五脏积热、壅毒、揩擦、疥痨之病。

胸堂穴：在膺骨两边[4]。是穴入针三分，出血。疗五脏积热，胸膊一切痛病。

带脉穴：在肘后四指[5]。是穴入针二分，出血。疗黑汗及[illegible]黄病。

督穴：在肾尖两边[6]，相对。是穴入针二分，出血。疗腰[illegible]滞气及肾脏风邪、把胯病。

尾本穴：在尾根底四指[7]。是穴入针三分，出血。疗脊间[illegible]气、把腰病。

同筋穴：在里乘重、膺骨下四指[8]。是穴入针一分。出血[illegible]疗闪擘着、乘重骨肿痛、并心疸病。

夜眼穴：在夜眼下[9]。是穴灸穴，禁止针刺。

曲池穴：在两后脚雁翅骨下，曲盘凹处[10]。是穴入针三分[illegible]出血，疗雁翅骨肿大及鹅鼻骨肿疼病。

膝脉穴：在膝下四指[11]，筋前骨后。是穴入针二分，出血[illegible]疗闪擘着、夹膝骨肿、皮骨劳、绞痛病[12]。

缠腕穴：在前攒筋骨上，后鹿节骨上，筋前骨后[13]。是穴[illegible]

针三分，出血。疗骨节肿痛，板筋肿病。封裹至效。

蹄头穴[14]：在前脚川字上，后脚八字上，共四穴。入针三分，出血。疗攒筋、鹿节骨肿疼，及蹄胎肿毒病，用药封裹。

里外项上共一十八[15]穴，疗马患项脊慪，低头不得病。

上上委、上中委、上下委、中上委、中中委、中下委、下上委、下中委、下下委。以上十八穴，入针一寸三分。疗项脊慪、低头不得。火针不效即烙。若是血气壅，即出胸堂、鹘脉大血。

……用火针于八窌穴，尾前短筋以后，两面共有六针，并八窌穴是七穴[16]，各去脊梁四指[17]。是穴入针二寸五分。若是冷风吹着，及檐下雨水淋着，或脏腑冷气传流，并内所伤，须灌补暖温脾气药及温熁[18]。若是损伤骨髓、肉断，血脉不通，须灌补暖血脉止痛药，并用火针。

膊上八穴：两面共一十六针，用火针各入一寸。疗血脉凝滞、肺气把膊、及膊尖骨肿大肿痛病[19]。膊尖、膊拦、冲天、抢风、肺门、肺攀、掩肘、乘镫。

弓子二穴[20]：在弓子骨上四指。是穴入针一寸三分，扳拽动皮，入温气。疗膊怯气滞病。

胯上八穴[21]：两面共一十六穴。火针各入针一寸。疗内肾积冷，气把胯病。巴山、路股、大胯、小胯、汗沟、仰瓦、邪气、牵肾。

腰上三穴[22]：两面共六穴，肾棚、肾俞、肾角，并接脊骨前百会一穴，共七穴。去脊梁四指，每穴相离四指。火针入一寸五分。疗内肾积冷，气把腰病。

肝俞穴：在左里仁畔[23]，从后第五肋里，去脊梁一尺五寸。是穴火针入一寸，疗一切肝家之病。

脾俞穴[24]：在从后第三肋里，自脊梁仰手却合手。是穴火针入一寸。疗脾胃伤冷、脾寒、打颤、脾不磨病。

肺俞穴：在从后第九肋里，去脊梁一尺五寸。是穴火针入一寸。疗肺气滞及肺痛病。

两耳中有禁穴二道，不得针刺。

风门穴[25]：在两耳根后面一指。是穴火烧烙铁圆烙。深三分，油涂。疗卒中[26]、破伤风或诸风病。

大风门穴三道[27]：在额上檐、睛鬃下。是穴火烧烙铁，烙深三分。疗肝昏、脑黄病。三穴一名。

通关穴二道：在舌根底下两边。是穴入针二分[28]，出血。疗六脉闭塞、舌本胀病。

玉堂穴：在口内上腭第三棱。是穴入针二分，出血，仍用盐搽之。疗五脏伏热、脑壅、束口黄病。

开关穴二道：在口内两颊上肿处。是穴火烧顶子[29]，烙二分，入盐搽，灌凉药。疗上焦壅热，咽水草难病。

喉门穴二道：在颊下一指相对。是穴病轻，即针通关[30]，各入三分；重即火烧烙铁铃圈[31]，疗骨胀紧硬，咽水草难病。

喉俞穴[32]：在颊下四指。是穴针钩，刀割开，眼圆二寸，透气。疗热壅呀呷，及束颡黄倒地病。

云门穴：在大马脐前三寸，小马二寸半。是穴火针入一寸。疗膀胱积冷、停留宿水病。

蹄门穴：在蹄两边。是穴火烧烙铁角，点烙出脓，油涂。疗蹄门并弹子头肿痛、点脚病。

天臼穴：在蹄门上窝子。是穴用粗火针入三分。疗久患蹄病。

伏兔穴：在耳后二指。是穴入火针三分。疗项紧硬病。

骨眼穴：在眼内。先将针线穿过眼骨边头，[33]左手牢把线，右手用刀子割去骨眼，不许割着水轮。疗骨眼遮睛病，如果不割了，即眼不见物。

心俞穴：在臆骨上。是穴如患心疸黄病[34]，用白针镞十[illegible]针，针出黄水或血，将盐一钱搽在针处，拨出黄水毒气；如不[illegible]疗，成疮透心肺。

板筋穴：在膝下。是穴如患板筋大硬。用烙铁点烙。

鹿节骨穴：在鹿节骨上，筋前骨后。是穴入针二分，出血。疗失节肿痛病。

尾尖穴：在尾尖上。是穴针五分，出血。疗马黑汗病及痄尾病。

肚口穴：此穴通流小便，不许行针[⑤]。

膁癖穴：在软膁上。是穴火针三针，各入三分。疗膁垂紧硬或腹细病。

血堂穴：在两鼻内。是穴入针三分，出血。疗肺热攻注、鼻肿痛。

三江大脉穴：在鼻梁两边四指。是穴人针二分，出血。疗热气攻注、颊骨肿痛，或疗骨劳、绞痛病[⑥]。

垂睛穴：在眼上四指。是穴如患肿痛，毒气不散，白针镞之。

鬐中穴：在脊梁前高处。是穴如患一切肿痛，用白针镞，消散毒气。

掠草穴：在曲池上。是穴入火针三分，针一名[⑦]。疗腿牵拽胯病。

锁口穴：在口角两面。是穴如患锁口黄病，用烙铁烙。深三分，长一寸半。

外乘重穴：在膝上五寸。是穴入火针三分。疗闪着、肿毒、或脏腑攻注肿痛病。

垂泉穴：在蹄底雀舌。是穴用尖顶烙深三分[⑧]。疗久患蹄漏、肿痛、毒气不出病。

阴俞穴：在外肾后中心缝上[⑨]。是穴火烧钉子烙，深三分，用油涂。疗阴肾肿大并木肾病。

医马买马吉日，不拘月份用之：

己巳　庚午　辛未　乙亥　丙子　丁丑　乙酉　丁亥　戊子　壬辰　乙未　戊戌　乙亥　辛丑　甲辰　乙巳　丙午　己酉　壬

子　丁巳　戊午　己未　庚申　壬戌

放血忌日：

春寅午戌　夏巳酉丑　秋申子辰　冬亥卯未

马本命日：

九月巳日　十月亥日　十一月午日　十二月子日

以上日不宜行针医治。欲行针，如犯血忌、本命、晦朔弦望、风雨阴寒，皆是禁忌，不可行针。又缘春首及马有病，弃血如泥；余月及马无病，惜血如金。凡针马之疾，先观马之肥瘦，次看吃草多少，然后相度行之；针皮不得伤肉，针肉不得伤筋、伤骨。三补一泻。大马先针左，骒马先针右，后学者识之。

[注释]

① 伯乐针经是假托伯乐的作品，《隋书·经籍志》中尚无此书名。新旧《唐书》艺文志、经籍志中无兽医书目录及文献记载。《宋史·艺文志》中始见此书名，因其收录在《安骥集》卷一中，证明伯乐针经是唐代作品。

② 眼脉穴在眼外角4.5厘米的面横静脉上，二穴。善泻肝经之火，用治眼结膜炎、角膜炎，肝经风热，脑充血，脑炎，月盲眼，中暑等。

③ 颊骨，指下颌骨。鹘脉穴在马颈静脉沟上、中三分之一交界处的颈静脉上，出血量视病情及马体大小肥瘦而定。泻血热，消黄肿，退热消炎[illegible]用治遍身黄、热性病、心热风邪、急性中毒、肺炎、脑充血等。

④ 臆骨，指胸骨前部。胸堂穴位于胸前两侧，胸外静脉沟下部，桡[illegible]上端水平位置处，小宽针刺在皮下臂头静脉上，疗心经积热、胸膊一切[illegible]痛病、肺气把膊病、肺把低头难等病。

⑤ 带脉穴，在肘突后胸侧壁的胸外静脉上，治肠炎、冷痛、中暑等症。

⑥ 督穴，在后肢内侧肾堂穴下6厘米处的隐静脉上。主治风伤腰胯[illegible]腰胯风湿、五攒痛、外肾黄等。督穴、肾堂穴和交当穴三穴位于同一的[illegible]静脉上，治的疾病相同，同是放血穴，在治疗上只选用一穴，须二次[illegible]时更换一穴即可。

⑦ 尾本穴，在尾根腹侧正中，针刺皮下尾静脉出血，疗闪伤腰胯，[illegible]气把腰胯和后躯风湿、尾神经麻痹、肠炎、泌尿系统疾病。
