# Supplementary material for: Historical Facts of Acupuncture and Traditional Chinese Veterinary Medicine—A Letter to the Editor Re: Magalhães-Sant’Ana, M. Animals 2019, 9, 168
Source: Animals (Basel). 2020 Jul 15;10(7):1196. doi: 10.3390/ani10071196 (PMC7401525; doi:10.3390/ani10071196)
Supplement: Supplementary file 1 [file animals-10-01196-s001.zip › Supplementary materials/PDF2 Bo Le Ming Tang Lun.pdf]

元亨疗马集校注

丁宾序本

于　船　郭光纪　郑动才　李德福　校注

北京农业大学出版社

责任编辑　雷克敬
插　　图　荆允正　雷克敬

元亨疗马集校注

于　船　郭光纪　郑动才　李德福　校注

*

北京农业大学出版社出版发行
（北京市海淀区圆明园西路）
北京外文印刷厂印刷

新　华　书　店　经　销

*

32开本　850×1168毫米　21印张　530千字
1990年12月第1版　1990年12月第1次印刷
印数：1—3000册

ISBN 7-81002-090-0/S·91
定价．22.00元（平装）
28.00元（精装）

伯乐明堂(1)论

秦穆公(2)问于伯乐曰："马于春首针刺出血者，何谓也？"伯乐答曰："人受气于癸(3)，癸者，阴水也；水主肾，肾主精，故精气多，而血气少。马受气于丙(4)，丙者，阳火也(5)；火主心，心主血，故血气多，而精气少。故马必于针刺出血者，不使血气太盛而为病疾也。"公曰(一)："出血必于春首者，何也？"伯乐答曰："盖春者，木也；夏者，火也；木火相生也。马为火畜，生于寅(二)(6)，旺于午(7)，伏于戌(8)；且寅宫有丙火(9)，午宫有丁火(10)，戌宫亦有阴火也(11)；于春首针刺，分调血气，不致太盛，故使火畜至夏，夏季火炎，使其营卫调匀，不致遥过而生诸疾也。"穆公曰："善。余闻牛马周身有一十二道经脉(12)，三百六十道络脉(13)者，何也？"答曰："经脉者，血筒也；络脉者，穴(三)孔(14)也。"穆公曰："愿卒闻之。"伯乐答曰："凡针血穴者，必须先于明堂经内，穷究血道穴(四)孔，推详所治之则，然后观其天气晴静，及月令盈虚(15)晦朔弦望(16)，与夫本命、刀砧(17)、血支(18)、血忌(19)、风雨阴寒，皆为禁忌，不可妄施针烙。此谓用针之致也。昔黄帝问于师皇曰："用针之道有法则焉？"师皇对曰："法天则地，合以天光日月也。"帝曰："愿闻其道。"师皇对曰："针者，有揭病之功；刺者，须当应病。但于四时气正而治，无不应效也。气正者，晴明也，光霁也，温和也；使其马牛气血调和，而卫气(20)易行、荣气(21)易泄也。是故，天寒无刺(22)，天温无凝(五)(23)，月生无彻，月满无补，月缺无泻，月晦无针，月空无治(24)。此谓应天时，合月令，黄帝、师皇用针之要也。"穆公曰："应病行针，其有道乎？"曰："有。""其妙几何？"伯乐答曰："凡用针者，必须谨敬严肃，当先令兽停立宁静，喘息调匀，右手持(六)针，左手按穴，量用锋头大小，及观马之身体肥瘦，食草多

少，察其寒热盛衰，然后方可施针。针皮勿令伤肉，针肉勿令伤筋[七]、伤骨。隔一毫，如隔泰[八]山；偏一丝，不如不针。三补一泻[25]。大马先针左，骒[九]马先针右；从阴引阳[26]，从阳引阴[27]；以右治左，以左治右[28]。如针六脉出血，升合多寡，及验血之荣悴，分调太过不及，此谓用针之道也。”穆公曰：“善。”又曰：“夫兽者，虽有刍水，周身有三斗六升血气，有三百六十骨节，亦有三百六十穴道。凡在医者，必须察其虚实，审其轻重，明其表里，度其浅深。大抵用针之道，虚之则补，实之则泻，寒之则温，热之则凉，风之则散，气之则顺。此谓一定之法，学者诚心鉴之。”

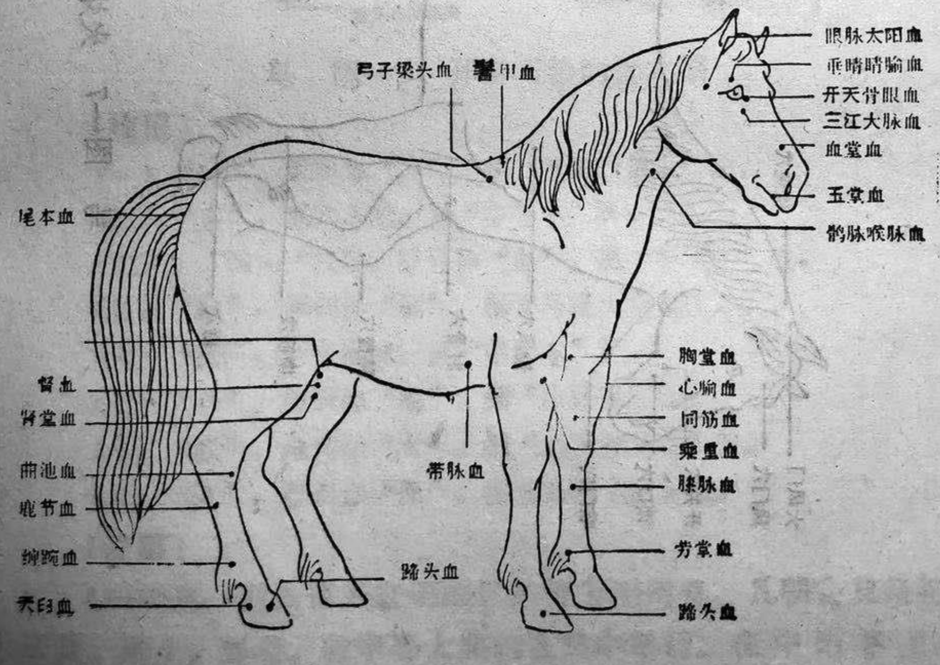

马图—6　六脉出血明堂之图

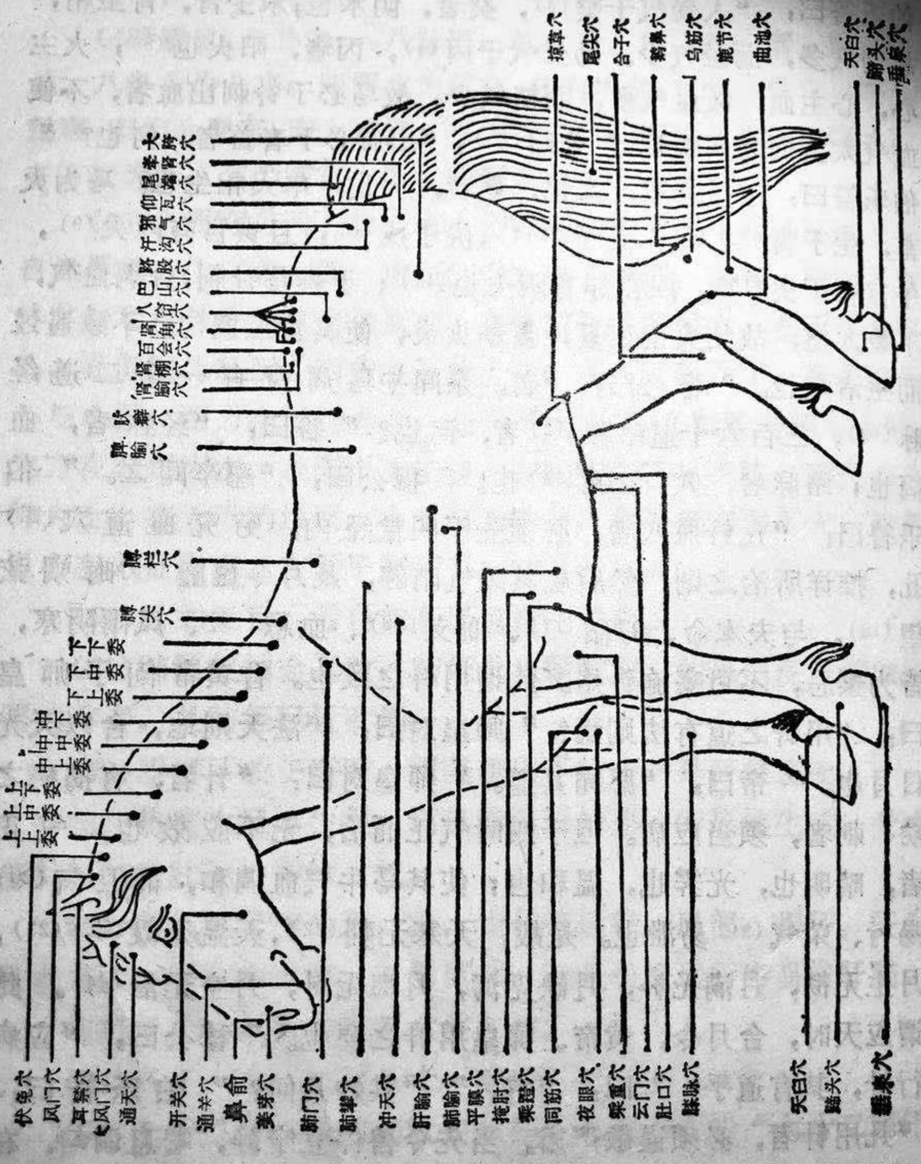

马 图—5 火针气针明堂之图
