# Supplementary material for: Historical Facts of Acupuncture and Traditional Chinese Veterinary Medicine—A Letter to the Editor Re: Magalhães-Sant’Ana, M. Animals 2019, 9, 168
Source: Animals (Basel). 2020 Jul 15;10(7):1196. doi: 10.3390/ani10071196 (PMC7401525; doi:10.3390/ani10071196)
Supplement: Supplementary file 1 [file animals-10-01196-s001.zip › Supplementary materials/PDF3 Liu Mai Ming Tang Ge.pdf]

元亨疗马集校注

丁宾序本

于 船 郭光纪 郑动才 李德福 校注

北京农业大学出版社

责任编辑　雷克敬
插　　图　荆允正　雷克敬

元亨疗马集校注

于　船　郭光纪　郑动才　李德福　校注

*

北京农业大学出版社出版发行
（北京市海淀区圆明园西路）
北京外文印刷厂印刷

新　华　书　店　经　销

*

32开本　850×1168毫米　21印张　530千字
1990年12月第1版　1990年12月第1次印刷
印数：1—3000册

ISBN　7-81002-090-0/S·91
定价．22.00元（平装）
28.00元（精装）

六脉明堂歌

凡三百六十句　按三百六十穴

盖天之生物[1]，有物而有则[2]；
马牛之为物，富民而利国；
耒耜[3]教天下，弧矢镇边阙[4]，
平戎而定寇[5]，务滋而稼穑[6]。
乘之与驾之，伤寒与伤热，
走骤失调和，饮喂失时刻，
疴瘵渐萌生，匪医而弗克。
近之愚学者，伪能者广阔，
以其衣冠美，巧言而令色[7]；
不审其根源，不察其色脉；
记诵邪口词[8]，诬民而世惑[9]。
妄施针与烙(一)，痞(二)哑难分(三)说[10]。
缪辈[11]之流传，万古而充(四)塞；
呜呼横夭(五)多，予心悼之切！
采摭[12]师皇论，诸贤之简策[13]，
有同而有异，咏成歌与诀。
针烙有所施，疾病有所揭[14]，
读者易于心，开发童蒙惑；
继先之准绳，传后之法则，
予愚不敏矣，僭逾[15]罪难越；
以俟后之能，削赘补失阙[16]。
六脉明堂歌，载之于后列。
眼脉鹘脉血，胸堂并带脉，

肾堂及尾本，同筋夜眼穴，
缠腕及蹄头，曲尺膝脉节，
都来十一针，名为六脉血。
用针须用意，按典依经说：
针皮针血筒，勿伤筋骨节；
隔丝如隔山，偏较(17)不见血；
此法要精通，非通勿妄泄；
一百五十九，不出十一血；
学者致精微，须当用意彻。
六脉外有针，逐一与君说：
玉堂与血堂，三江大脉血，
脏热鼻梁肿，此血须当彻。
口角连腮肿，火烙锁(六)口穴。
起卧肠中痛，剜取双姜节。
肝热闪(七)骨生，线穿骨眼穴。
开天取浑睛；垂睛医眼热。
风症烙风门，更兼伏兔穴。
束颡及三喉，喉腧开喉节。
火烙(八)两喉门；颊下取槽结。
通关医舌胀；开关疗上热。
耳内有一针，禁穴休教彻。
项苓九)(18)针九委，两边十八穴。
心腧消黄疸；膝肿针膝脉。
肺把胸膊痛，肺门肺攀穴，
膊尖与膊栏，掩肘乘镫节，
抢风与冲(十)天，八穴火针彻；
两边十六针，滞气俱消灭。
拽皮入温气，白针弓子穴；

一切梁头(19)肿，髻(十一)甲几针泄。
肝腧治肝危，火针第五肋。
脾腧疗脾寒；肺腧肺痛彻。
云门放宿水，肚口(20)休教泄。
肾毒削垂泉，蹄修八字穴。
气把腰中痛，火针百会节；
肾棚并肾腧，肾角两边列，
七窍(21)总皆针，凝滞俱开彻。
脊痛吊腰行，尾本须针泄。
一切肾家壅，肾堂须出血。
督穴肾尖针，肾腰滞气绝。
肾抽胯瓦痛，牵连雁翅拽；
巴山与路股，大胯小胯穴，
邪气与汗(十二)沟，仰瓦牵肾穴，
八穴火针施，湿气皆消灭。
阴肾木肾肿，阴腧火针彻。
脏冷脱肛肠，剪取莲花穴。
行动尾根偏，尾端针尾节。
脊吝(十三)板如椽，火烙八窌穴。
拖脚掠草痛，掠草火针截。
缠腕消筋胀；同筋开膈(十四)热。
黑汗(十五)彻尾尖，肷癖医腹阔。
膈痛放胸膛；肠黄针带脉。
两目翳膜生，针取太(十六)阳穴。
久患蹄头痛，火针天臼(十七)节。
鹅鼻曲池肿，曲池须针泄。
攒筋板筋粗，火烙板筋节。
失节肿痛病，鹿(十八)节须当彻。

掌骨痛难行，火烙蹄门穴。
乘重骨(十九)肿痛，还将乘重彻。
黄疸布胸痈，心腧几针泄。
尾本治腰风，擦尾燥鬃绝。
项吝兼脊吝(22)，火针三委节(23)。
三喉显大功，莫过喉腧穴。
肾热彻交当，腰间滞气灭。
劳堂(二十)泻少阳，筋胀从斯(二十一)绝。
松骨肿如肥，血堂兼大脉。
带脉疗肠黄，黑汗偏能揭。
蹄痛放蹄头，筋毒能消泄。
草慢嚼(二十二)不鸣，盐擦玉堂血。
睛明大眥(二十三)针，赤脉眼中灭。
心热肿偏次，胸堂能冷泄。
毛燥若虫行，鹘脉两针绝。
倒地损抢风，火烙抢风穴。
昂头点步行，膊尖温火截。
直行膝盖疼，膝脉须针彻。
千金騸肾囊(24)，蹄啮俱能灭。
气海鼻头开，善泄心胸热。
抽(二十四)筋唇上挑，走骤俱开阔。
草谷不消磨，火针脾腧穴。
乌金合骨胪(二十五)，烙之合骨节。
牵拽雁翅疼，大胯火能绝。
蹄门消蹄肿，尾尖(二十六)疳尾彻。
肷黄(二十七)放肾堂；肘痈泄带脉。
牵肾攻抽肾；鹿节针失节。
肾腧治腰疼，汗沟医胯拽。

一切梁头(19)肿，髻(十一)甲几针泄。
肝腧治肝危，火针第五肋。
脾腧疗脾寒；肺腧肺痛彻。
云门放宿水，肚口(20)休教泄。
肾毒削垂泉，蹄修八字穴。
气把腰中痛，火针百会节；
肾棚并肾腧，肾角两边列，
七窍(21)总皆针，凝滞俱开彻。
脊痛吊腰行，尾本须针泄。
一切肾家壅，肾堂须出血。
督穴肾尖针，肾腰滞气绝。
肾抽胯瓦痛，牵连雁翅拽；
巴山与路股，大胯小胯穴，
邪气与汗(十二)沟，仰瓦牵肾穴，
八穴火针施，湿气皆消灭。
阴肾木肾肿，阴腧火针彻。
脏冷脱肛肠，剪取莲花穴。
行动尾根偏，尾端针尾节。
脊宫(十三)板如椽，火烙八窌穴。
拖脚掠草痛，掠草火针截。
缠腕消筋胀；同筋开膈(十四)热。
黑汗(十五)彻尾尖，肷癖医腹阔。
膈痛放胸膛；肠黄针带脉。
两目翳膜生，针取太(十六)阳穴。
久患蹄头痛，火针天臼(十七)节。
鹅鼻曲池肿，曲池须针泄。
攒筋板筋粗，火烙板筋节。
失节肿痛病，鹿(十八)节须当彻。

邪气去风寒。玉堂消脏热。
鹘脉(二十八)太阴经，一名号大血，
寒热善能医，定神而安魄。
耳根去一指，风门两道穴。
二指烙伏兔，顶门三道截。
七窍(25)号风关，火烙诸风灭。
腰上有一针，名为百会穴，
火烙此千津(26)，后腰风尽绝。
春来万病生，大血(二十九)两针彻，
诸毒不能成，百病俱消灭。
以上几般针，疴瘵从斯揭。
量度浅(三十)深施，辨验阴阳彻。
针血有阴阳，经络十二诀，
医工仔细详，须当再与说。
眼脉厥阴经，肝火善能泄。
鹘脉肺太阴，能消五脏热。
胸堂少阴心，刺之心火绝。
夜眼禁其针，厥阴心包脉。
带脉泻太阴，脾火尽消灭。
肾热肾经肿，肾堂少阴彻。
少阳三焦壅，须泄蹄头血。
膝脉大肠(三十一)经，善解阳明热。
缠腕号劳堂，少阳肝胆泄。
阳明胃火消，曲池两针血。
膀胱泻太阳，尾本尾根彻。
同筋泻小肠，太阳心火绝。
此法甚玄微，非精莫妄彻。
凡针血与穴，医家用意窃；

穷理(27)阴阳病，观诊色与脉，
行步相安详，喘息听时节；
表里与邪正，虚实与寒热，
较斟轻与重，度量浅深彻，
针烙按明堂，百无一二绝。
近学晚医人，凡针六脉血(三十二)，
不在苦令多(28)，四季相应彻；
春夏脉洪弦，秋冬脉沉濇，
春彻更无妨，冬针膘瘦怯，
有疾弃如泥，无疴金惜血，
先观(三十三)肥与瘦，次辨寒与热，
食草多与少，审察方针彻。
彻毕休教饮，饮毕休教彻，
彻后休教浸，最忌水中涉。
牵行似醉痴，舌如煮豆色，
见此莫行针，血出无休歇。
三百六十针，百五十九穴，
四百八病中，大半(三十四)针工揭。
寒甚火针施，热甚生针彻；
急病急须针，勿差时与刻。
救疗有功能，莫过针与穴。
针法有如斯，一一分明说，
学者莫蹉跎，须当用意窃；
察病察根源，观形观色脉，
彻血究尫羸，行针穷补泻，
非病莫行针，无伤血(三十五)莫彻，
冬血惜如金，春荣如水彻。
火针与气针，医工仔(三十六)细格(29)，

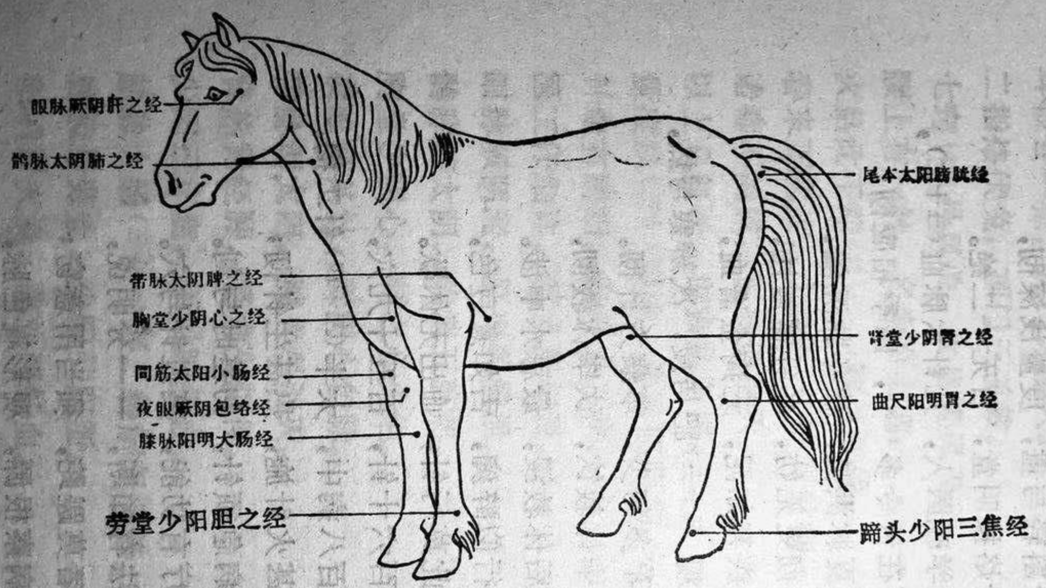
眼脉厥阴肝之经
鹘脉太阴肺之经
带脉太阴脾之经
胸堂少阴心之经
同筋太阳小肠经
夜眼厥阴包络经
膝脉阳明大肠经
劳堂少阳胆之经
尾本太阳膀胱经
肾堂少阴肾之经
曲尺阳明胃之经
蹄头少阳三焦经

马 图—9 三阴三阳之图

呼吸与提按，(30)，浅深与补泻，
右手持其针，左手按其穴，
天气要晴明，风雨须停歇。
针穴合天时，月令盈虚别。
月生血始精(31)，气血行诸节；
月圆肌肉坚，诸窍皆精脉；
月空经络虚，气衰脉部涩；
月满休教补，月缺休教泻，
月晦莫教针，窍关皆闭塞；
月令按阴阳，日时须拣择，
血忌与血支，飞廉兼命绝(32)，
本命及刀砧，遇(三十七)此休针彻。
左转针为补，右捻(三十八)针为泻，
或补须与补，当泻即须泻，
补泻要分明，非明勿浪说；
此法若能通，金锁都开彻，
治病显其功，有如汤浇雪；
学者要推寻，神功无净揭。

〔校记〕

(一) "烙"，原刊作"络"。据"中华本"改。
(二) "痞"，原刊作"瘖"。据"中华本"改。
(三) "分"，原刊作"食"。据"中华本"改。
(四) "充"，原刊作"克"。据"中华本"改。
(五) "夭"，原刊作"天"。据"中华本"改。
(六) "锁"，原刊作"镤"。据"善成堂许序本"改。
(七) "闪"，原刊作"闵"。据"中华本"改。
(八) "烙"，原刊作"络"。据"中华本"改。
(九) "吝"，原刊作"恡"。据"重编校正本"改。
