# Supplementary material for: Historical Facts of Acupuncture and Traditional Chinese Veterinary Medicine—A Letter to the Editor Re: Magalhães-Sant’Ana, M. Animals 2019, 9, 168
Source: Animals (Basel). 2020 Jul 15;10(7):1196. doi: 10.3390/ani10071196 (PMC7401525; doi:10.3390/ani10071196)
Supplement: Supplementary file 1 [file animals-10-01196-s001.zip › Supplementary materials/PDF4 Lun Ma Ming Tang Zhen Xue Zhe He Ye.pdf]

元亨疗马集校注

丁宾序本

于　船　郭光纪　郑动才　李德福　校注

北京农业大学出版社

责任编辑　雷克敬
插　　图　荆允正　雷克敬

元亨疗马集校注

于　船　郭光纪　郑动才　李德福　校注

*

北京农业大学出版社出版发行
（北京市海淀区圆明园西路）
北京外文印刷厂印刷

新　华　书　店　经　销

*

32开本　850×1168毫米　21印张　530千字
1990年12月第1版　1990年12月第1次印刷
印数：1—3000册

ISBN　7-81002-090-0/S·91
定价．22.00元（平装）
28.00元（精装）

六脉明堂歌

凡三百六十句　按三百六十穴

盖天之生物[1]，有物而有则[2]；
马牛之为物，富民而利国；
耒耜[3]教天下，弧矢镇边阙[4]，
平戎而定寇[5]，务滋而稼穑[6]。
乘之与驾之，伤寒与伤热，
走骤失调和，饮喂失时刻，
疴瘵渐萌生，匪医而弗克。
近之愚学者，伪能者广阔，
以其衣冠美，巧言而令色[7]；
不审其根源，不察其色脉；
记诵邪口词[8]，诬民而世惑[9]。
妄施针与烙(一)，瘖(二)哑难分(三)说[10]，
缪辈[11]之流传，万古而充(四)塞；
呜呼横夭(五)多，予心悼之切！
采摭[12]师皇论，诸贤之简策[13]，
有同而有异，咏成歌与诀。
针烙有所施，疾病有所揭[14]，
读者易于心，开发童蒙惑；
继先之准绳，传后之法则，
予愚不敏矣，僭逾[15]罪难越；
以俟后之能，削赘补失阙[16]。
六脉明堂歌，载之于后列。
眼脉鹘脉血，胸堂并带脉，

肾堂及尾本，同筋夜眼穴，
缠腕及蹄头，曲尺膝脉节，
都来十一针，名为六脉血。
用针须用意，按典依经说：
针皮针血筒，勿伤筋骨节；
隔丝如隔山，偏较(17)不见血；
此法要精通，非通勿妄泄；
一百五十九，不出十一血；
学者致精微，须当用意彻。
六脉外有针，逐一与君说：
玉堂与血堂，三江大脉血，
脏热鼻梁肿，此血须当彻。
口角连腮肿，火烙锁(六)口穴。
起卧肠中痛，剜取双姜节。
肝热闪(七)骨生，线穿骨眼穴。
开天取浑睛；垂睛医眼热。
风症烙风门，更兼伏兔穴。
束颡及三喉，喉腧开喉节。
火烙(八)两喉门；颊下取槽结。
通关医舌胀；开关疗上热。
耳内有一针，禁穴休教彻。
项吝九)(18)针九委，两边十八穴。
心腧消黄疸；膝肿针膝脉。
肺把胸膊痛，肺门肺攀穴，
膊尖与膊栏，掩肘乘镫节，
抢风与冲(十)天，八穴火针彻；
两边十六针，滞气俱消灭。
拽皮入温气，白针弓子穴；

一切梁头[19]肿，警[十一]甲几针泄。
肝腧治肝危，火针第五肋。
脾腧疗脾寒；肺腧肺痛彻。
云门放宿水，肚口[20]休教泄。
肾毒削垂泉，蹄修八字穴。
气把腰中痛，火针百会节；
肾棚并肾腧，肾角两边列，
七窍[21]总皆针，凝滞俱开彻。
脊痛吊腰行，尾本须针泄。
一切肾家壅，肾堂须出血。
督穴肾尖针，肾腰滞气绝。
肾抽胯瓦痛，牵连雁翅拽；
巴山与路股，大胯小胯穴，
邪气与汗[十二]沟，仰瓦牵肾穴，
八穴火针施，湿气皆消灭。
阴肾木肾肿，阴腧火针彻。
脏冷脱肛肠，剪取莲花穴。
行动尾根偏，尾端针尾节。
脊吝[十三]板如椽，火烙八窌穴。
拖脚掠草痛，掠草火针截。
缠腕消筋胀；同筋开膈[十四]热。
黑汗[十五]彻尾尖，肷癖医腹阔。
膈痛放胸膛；肠黄针带脉。
两目翳膜生，针取太[十六]阳穴。
久患蹄头痛，火针天臼[十七]节。
鹅鼻曲池肿，曲池须针泄。
攒筋板筋粗，火烙板筋节。
失节肿痛病，鹿[十八]节须当彻。

掌骨痛难行，火烙蹄门穴。
乘重骨（十九）肿痛，还将乘重彻。
黄疸布胸痈，心腧几针泄。
尾本治腰风，擦尾燥鬃绝。
项吝兼脊吝(22)，火针三委节(23)。
三喉显大功，莫过喉腧穴。
肾热彻交当，腰间滞气灭。
劳堂（二十）泻少阳，筋胀从斯（二十一）绝。
松骨肿如肥，血堂兼大脉。
带脉疗肠黄，黑汗偏能揭。
蹄痛放蹄头，筋毒能消泄。
草慢嚼（二十二）不鸣，盐擦玉堂血。
睛明大眥（二十三）针，赤脉眼中灭。
心热肿偏次，胸堂能冷泄。
毛燥若虫行，鹘脉两针绝。
倒地损抢风，火烙抢风穴。
昂头点步行，膊尖温火截。
直行膝盖疼，膝脉须针彻。
千金騸肾囊(24)，蹄啮俱能灭。
气海鼻头开，善泄心胸热。
抽（二十四）筋唇上挑，走骤俱开阔。
草谷不消磨，火针脾腧穴。
乌金合骨胪（二十五），烙之合骨节。
牵拽雁翅疼，大胯火能绝。
蹄门消蹄肿，尾尖（二十六）疳尾彻。
肷黄（二十七）放肾堂；肘痈泄带脉。
牵肾攻抽肾；鹿节针失节。
肾腧治腰疼，汗沟医胯拽。

一切梁头[19]肿，䯁[十一]甲几针泄。
肝腧治肝危，火针第五肋。
脾腧疗脾寒；肺腧肺痛彻。
云门放宿水，肚口[20]休教泄。
肾毒削垂泉，蹄修八字穴。
气把腰中痛，火针百会节；
肾棚并肾腧，肾角两边列，
七窍[21]总皆针，凝滞俱开彻。
脊痛吊腰行，尾本须针泄。
一切肾家壅，肾堂须出血。
督穴肾尖针，肾腰滞气绝。
肾抽胯瓦痛，牵连雁翅拽；
巴山与路股，大胯小胯穴，
邪气与汗[十二]沟，仰瓦牵肾穴，
八穴火针施，湿气皆消灭。
阴肾木肾肿，阴腧火针彻。
脏冷脱肛肠，剪取莲花穴。
行动尾根偏，尾端针尾节。
脊斋[十三]板如椽，火烙八窌穴。
拖脚掠草痛，掠草火针截。
缠腕消筋胀；同筋开膈[十四]热。
黑汗[十五]彻尾尖，肷癖医腹阔。
膈痛放胸膛；肠黄针带脉。
两目翳膜生，针取太[十六]阳穴。
久患蹄头痛，火针天臼[十七]节。
鹅鼻曲池肿，曲池须针泄。
攒筋板筋粗，火烙板筋节。
失节肿痛病，鹿[十八]节须当彻。

邪气去风寒。玉堂消脏热。
鹘脉（二十八）太阴经，一名号大血，
寒热善能医，定神而安魄。
耳根去一指，风门两道穴。
二指烙伏兔，顶门三道截。
七窍（25）号风关，火烙诸风灭。
腰上有一针，名为百会穴，
火烙此千津（26），后腰风尽绝。
春来万病生，大血（二十九）两针彻，
诸毒不能成，百病俱消灭。
以上几般针，疴瘵从斯揭。
量度浅（三十）深施，辨验阴阳彻。
针血有阴阳，经络十二诀，
医工仔细详，须当再与说。
眼脉厥阴经，肝火善能泄。
鹘脉肺太阴，能消五脏热。
胸堂少阴心，刺之心火绝。
夜眼禁其针，厥阴心包脉。
带脉泻太阴，脾火尽消灭。
肾热肾经肿，肾堂少阴彻。
少阳三焦壅，须泄蹄头血。
膝脉大肠（三十一）经，善解阳明热。
缠腕号劳堂，少阳肝胆泄。
阳明胃火消，曲池两针血。
膀胱泻太阳，尾本尾根彻。
同筋泻小肠，太阳心火绝。
此法甚玄微，非精莫妄彻。
凡针血与穴，医家用意窃；

穷理(27)阴阳病，观诊色与脉，
行步相安详，喘息听时节；
表里与邪正，虚实与寒热，
较斟轻与重，度量浅深彻，
针烙按明堂，百无一二绝。
近学晚医人，凡针六脉血(三十二)，
不在苦令多(28)，四季相应彻；
春夏脉洪弦，秋冬脉沉濇，
春彻更无妨，冬针膘瘦怯，
有疾弃如泥，无疴金惜血，
先观(三十三)肥与瘦，次辨寒与热，
食草多与少，审察方针彻。
彻毕休教饮，饮毕休教彻，
彻后休教浸，最忌水中涉。
牵行似醉痴，舌如煮豆色，
见此莫行针，血出无休歇。
三百六十针，百五十九穴，
四百八病中，大半(三十四)针工揭。
寒甚火针施，热甚生针彻；
急病急须针，勿差时与刻。
救疗有功能，莫过针与穴。
针法有如斯，一一分明说，
学者莫蹉跎，须当用意窃；
察病察根源，观形观色脉，
彻血究尫羸，行针穷补泻，
非病莫行针，无伤血(三十五)莫彻，
冬血惜如金，春荣如水彻。
火针与气针，医工仔(三十六)细格(29)，

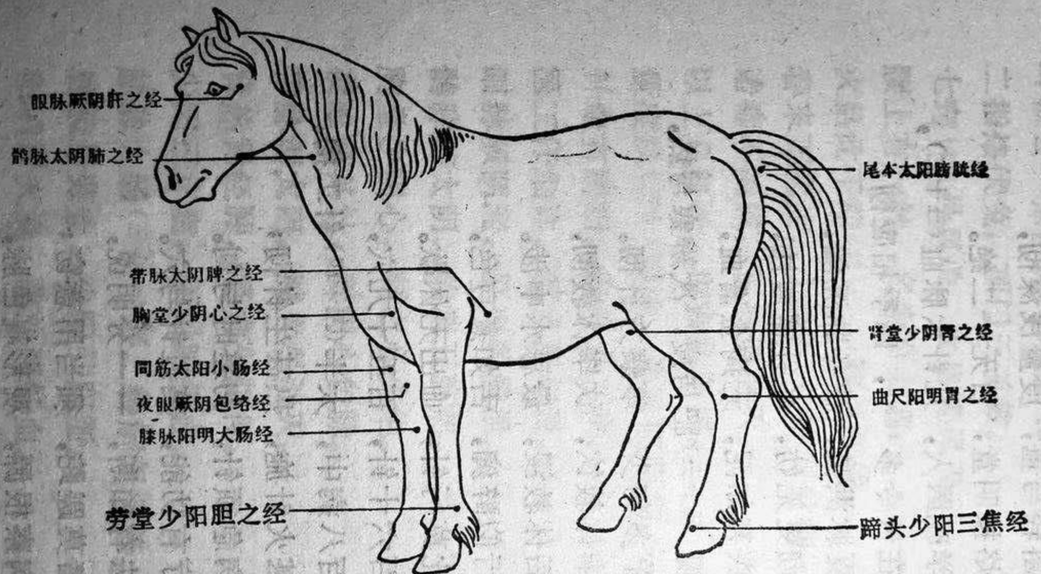
眼脉厥阴肝之经
鹘脉太阴肺之经
带脉太阴脾之经
胸堂少阴心之经
同筋太阳小肠经
夜眼厥阴包络经
膝脉阳明大肠经
劳堂少阳胆之经
尾本太阳膀胱经
肾堂少阴肾之经
曲尺阳明胃之经
蹄头少阳三焦经

马　图—9　三阴三阳之图

呼吸与提按，(30)，浅深与补泻，
右手持其针，左手按其穴，
天气要晴明，风雨须停歇。
针穴合天时，月令盈虚别。
月生血始精(31)，气血行诸节；
月圆肌肉坚，诸窍皆精脉；
月空经络虚，气衰脉部涩；
月满休教补，月缺休教泻，
月晦莫教针，窍关皆闭塞；
月令按阴阳，日时须拣择，
血忌与血支，飞廉兼命绝(32)，
本命及刀砧，遇(三十七)此休针彻。
左转针为补，右捻(三十八)针为泻，
或补须与补，当泻即须泻，
补泻要分明，非明勿浪说；
此法若能通，金锁都开彻，
治病显其功，有如汤浇雪；
学者要推寻，神功无净揭。

〔校记〕

(一)“烙”，原刊作“络”。据“中华本”改。
(二)“瘖”，原刊作“瘖”。据“中华本”改。
(三)“分”，原刊作“食”。据“中华本”改。
(四)“充”，原刊作“克”。据“中华本”改。
(五)“夭”，原刊作“天”。据“中华本”改。
(六)“锁”，原刊作“镤”。据“善成堂许序本”改。
(七)“闪”，原刊作“阕”。据“中华本”改。
(八)“烙”，原刊作“络”。据“中华本”改。
(九)“吝”，原刊作“恡”。据“重编校正本”改。
